# Anti-Convulsant Activity of *Boerhaavia diffusa*: Plausible Role of Calcium Channel Antagonism

**DOI:** 10.1093/ecam/nep192

**Published:** 2011-01-09

**Authors:** Mandeep Kaur, Rajesh Kumar Goel

**Affiliations:** Department of Pharmaceutical Sciences and Drug Research, Punjabi University, Patiala, Punjab Pharmacology division 147002, India

## Abstract

“Ethnopharmacological” use of roots of *Boerhaavia diffusa* (*B. diffusa*) in the treatment of epilepsy in Nigerian folk medicine and reports showing the presence of a calcium channel antagonistic compound “liriodendrin” in its roots, led us to undertake the present study. The study was designed to investigate the methanolic root extract of *B. diffusa* and its different fractions including liriodendrin-rich fraction for exploring the possible role of liriodendrin in its anti-convulsant activity. Air-dried roots of *B. diffusa* were extracted with methanol by cold maceration. The methanol soluble fraction of extract thus obtained was successively extracted to obtain liriodendrin-rich fraction and two side fractions, that is, chloroform fraction and phenolic compound fraction. Anti-convulsant activity of methanolic extract (1000, 1500 and 2000 mg kg^−1^, intraperitoneally (i.p.)) and its different fractions, that is, liriodendrin-rich fraction (10, 20 and 40 mg kg^−1^, i.p., chloroform fraction (20 mg kg^−1^, i.p.) and phenolic compound fraction (1 mg kg^−1^, i.p.) were studied in pentylenetetrazol (PTZ)-induced seizures (75 mg kg^−1^, i.p.). The crude methanolic extract of *B. diffusa* and only its liriodendrin-rich fraction showed a dose-dependent protection against PTZ-induced convulsions. The liriodendrin-rich fraction also showed significant protection against seizures induced by BAY k-8644. These findings reiterated the anti-convulsant activity of methanolic extract of *B. diffusa* roots. Furthermore, it can be concluded that the observed anti-convulsant activity was due to its calcium channel antagonistic action as this activity was retained only in the liodendrin-rich fraction, which has additionally been confirmed by significant anti-convulsant activity of liriodendrin-rich fraction in BAY k-8644-induced seizures.

## 1. Introduction

Ayurveda is a native Indian healthcare system which is currently used by millions of people in India, Nepal and Sri Lanka for their day-to-day healthcare needs [1]. *Boerhaavia diffusa* Linn. (Nyctaginaceae), commonly known as *punarnava* in Sanskrit, is a creeping weed found abundantly all over India. It has a long history of use by indigenous and tribal people [2]. In traditional system of medicine, the roots of *Boerhaavia diffusa* (*B. diffusa*) have been widely used for the treatment of dyspepsia, jaundice, spleenomegaly, abdominal pain, abdominal tumors and some other types of cancers [3]. In *Charaka Samhita* and *Sushrita Samhita*, it is mentioned that the Ayurvedic preparations made from *punarnava*—namely, *punarnavastaka kvath*, *punarnava kshar* and *punarnava taila*—were used for the treatment of various ailments such as stomachache, anemia, cough and cold, and used as laxative and expectorant. In Nigerian folk medicine it has been widely used for the treatment of epilepsy [4]. Many traditional uses of *B. diffusa* as, diuretic [5], anti-fibrinolytic [6], anti-bacterial [7], hepatoprotectant [8], anti-helmintic, febrifuge, anti-leprotic, anti-asthmatic, anti-scabies, anti-urethritis [9], anti-convulsant [10], cardiotonic [11], immunosuppressant, anti-viral and anti-oxidant [12], anti-inflammatory [13], anti-diabetic [14] and anti-cancer [15], had been validated pharmacologically.

*Boerhaavia diffusa* contains a large number of phytoconstituents namely, flavonoids, alkaloids, steroids, triterpenoids, lipids, lignins, carbohydrates, proteins, glycoproteins, punarnavine, boeravinones A–F, hypoxanthine 9-l-arabinofuranoside, ursolic acid, punarnavoside, punarnavoside and liriodendrin [12]. Liriodendrin was isolated from the methanolic extract of roots of *B. diffusa* and it showed a calcium channel antagonistic activity [16, 17]. Reports demonstrating the use of roots of *B. diffusa* in the treatment of epilepsy [10] and the presence of calcium channel antagonistic compound “liriodendrin” in its roots led us to hypothesize that, roots of *B. diffusa* may possess anti-convulsant activity due to calcium channel antagonistic effect of liriodendrin.

Hence the present study was designed to investigate different fractions of methanolic root extract of *B. diffusa* for exploring possible role of liriodendrin in its anti-convulsant activity.

## 2. Methods

### 2.1. Collection of Plant Material

*Boerhaavia diffusa* roots were collected from the botanical garden of Punjabi University, Patiala, India. The botanical identity of the plant material was verified and specimens were deposited at the Herbarium, Department of Botany, Punjabi University, Patiala, Punjab, for reference (Voucher No.: 50973).

### 2.2. Preparation of Liriodendrin-Rich Extract

**Figure 1:**
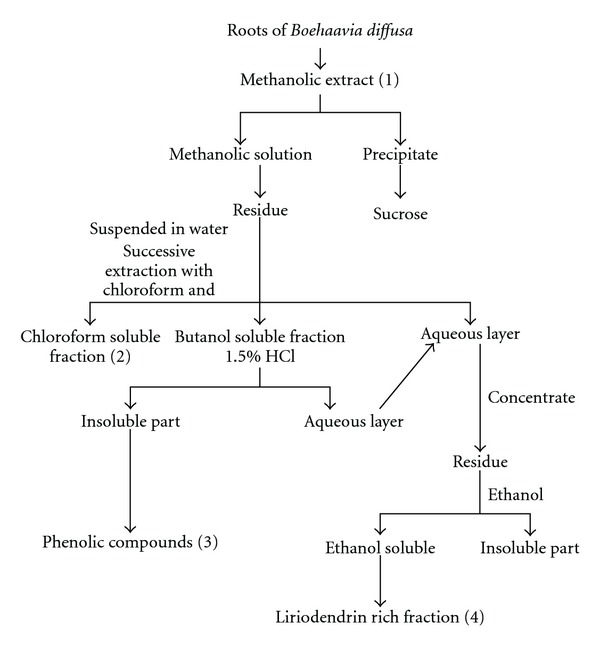
Scheme for extraction of roots of *B. diffusa*.

Authenticated roots were shade-dried, grounded to a moderately coarse powder and were extracted as per the scheme given in Figure 1 [17]. Briefly, the dried powdered roots of the plant (300 g) were subjected to extraction with 500 mL of methanol thrice by cold extraction. The resultant methanolic extract was pooled and evaporated to dryness using rotavapor. The crude methanolic extract (32 g) (fraction 1) thus obtained was again dissolved in methanol (about 150 mL) and the insoluble precipitate was separated by filtration which was found to be sucrose by Fehling's; test. The methanolic filtrate thus obtained was again evaporated to dryness using rotavapor and the residue thus obtained was suspended in water and fractionated by successive extraction with chloroform and butanol to give the chloroform (fraction 2), butanol and water fraction. The butanol fraction was extracted with 1.5% aqueous hydrochloric acid at room temperature and the insoluble material, which contained a mixture of phenolic compounds (fraction 3), was separated by filtration and the aqueous layer obtained from butanol fraction was pooled with aqueous layer obtained from methanolic solution. This pooled aqueous fraction was evaporated to dryness using rotavapor under reduced pressure. The residue thus obtained was dissolved in ethanol and the insoluble material was separated by filtration. The ethanolic solution was evaporated to dryness using rotavapor, giving the liriodendrin-rich fraction (fraction 4). The different fractions (1, 2, 3 and 4) were stored at 4°C and the fractions of methanolic extracts were administered at a dose equivalent to the effective dose of crude methanolic extract based on percentage yield of each fraction. Dose of the extract and its fractions was prepared by dissolving a weighed quantity of extract and fractions in dimethylsulfoxide (DMSO) and then dispersing the resultant solution in 0.5% carboxymethylcellulose (CMC) suspension (DMSO 1: CMC 9) freshly before use and was injected intraperitoneally (i.p.). Control groups received equal volume of vehicle (DMSO 1: CMC 9) (i.p.).

### 2.3. Drugs and Chemicals

All standard chemicals used in the present study were of analytical grade. Pentylenetetrazol (PTZ) was obtained from Sigma Chemical Company (USA), Diazepam from Jackson Laboratories Ltd (Amritsar, India), DMSO from Qualigens Fine Chemicals (Mumbai, India), CMC and Methanol from S.D Fine Chem Ltd (Mumbai, India) and BAY k-8644 from (Sigma-Aldrich, USA).

### 2.4. Animals

Male Swiss albino mice (*Mus musculus*), weighing 25–35 g obtained from Central Research Institute Kausali, Himachal Pradesh, were used in the present study. The animals were housed in standard cages and maintained at room temperature with natural day and night cycles. The animals were allowed free access to food (standard laboratory diet) and water during study. All experiments were carried out between 07:00 and 16:00 h. All procedures were conducted according to CPCSEA guidelines and approved by the Institutional Animal Ethical Committee.

### 2.5. Preliminary Phytochemical Screening

Crude methanolic extract of *B. diffusa* and its fractions 2, 3 and 4 were reconstituted in methanol to obtain 1% w/v stock solutions, which were subjected to preliminary phytochemical screening for the presence of alkaloids (Hager's test, Wagner's test), carbohydrates (Molisch's test), reducing sugars (Fehling's test), saponins (Foam test), tannins (FeCl3 test), flavonoids (Alkali test), phenolic compounds (Ferric chloride test) and proteins (Biuret test) [18].

### 2.6. PTZ-Induced Convulsions

Animals were divided into 12 different groups (*n* = 6). Vehicle, methanolic extract and different fractions of extract at specified doses were administered 30 min before induction of convulsions. Convulsions were induced by intraperitoneal injection of PTZ (75 mg kg^−1^). The animals were divided into different groups as follows:

**Group 1**

control group (vehicle treated).

**Group 2**

standard group (Diazepam 4 mg kg^−1^, i.p.).

**Group 3**

diazepam (1 mg kg^−1^, i.p.).

**Group 4**

crude extract (fraction 1) (1000 mg kg^−1^, i.p.).

**Group 5**

crude extract (fraction 1) (1500 mg kg^−1^, i.p.).

**Group 6**

crude extract (fraction 1) (2000 mg kg^−1^, i.p.).

**Group 7**

chloroform fraction (fraction 2) (20 mg kg^−1^, i.p.);

**Group 8**

phenolic compound fraction (fraction 3) (1 mg kg^−1^, i.p.).

**Group 9**

liriodendrin-rich fraction (fraction 4) (10 mg kg^−1^, i.p.).

**Group 10**

liriodendrin-rich fraction (fraction 4) (20 mg kg^−1^, i.p.).

**Group 11**

liriodendrin-rich fraction (fraction 4) (40 mg kg^−1^, i.p.).

**Group 12**

liriodendrin-rich fraction (fraction 4) (20 mg kg^−1^, i.p.) +Diazepam (1 mg kg^−1^, i.p.).

In all groups the latency to clonic convulsions and mortality was noted [19]. The severity of convulsions was scored as follows: Each phase was given a numeric score: unresponsiveness = 0, mild contractions = 1, clonic seizures = 2, tonic seizures = 3 and death = 4. The response of each animal was scored according to the highest phase reached and mean severity score was calculated for each group [20]. All the treated groups were compared with vehicle treated controls respectively in order to determine the significant anti-convulsant activity.

### 2.7. BAY k-8644-Induced Seizures

In order to elucidate the calcium channel antagonistic action of liriodendrin-rich fraction, animals were divided into four different groups (*n* = 6). Vehicle and liriodendrin-rich fraction (10, 20 and 40 mg kg^−1^) were administered 30 min prior to the intracerebroventricular injection of BAY k-8644 (37.5 *μ*g). Seizures induced were rated according to the scale devised by: stage 1, scratching and twisting of the forelimbs (score 1); stage 2, rearing and walking (score 2); stage 3, intermittent clonic jerks of limbs with tonic flexion of fore limbs and tail flexion; stage 4, head bobbing with complex grooming actions (licking of fur and scratching); stage 5, jumping, squeaking and tonic extension of hind limbs; stage 6, barrel rolling. The occurrence of clonic and tonic seizure signs and their latency were recorded for 50 min. Mortality in the animals was observed for 8 h after BAY k-8644 injection [21]. The animals were divided into different groups as follows:

**Group 13**

control group (vehicle-treated).

**Group 14**

liriodendrin-rich fraction (fraction 4) (10 mg kg^−1^, i.p.).

**Group 15**

liriodendrin-rich fraction (fraction 4) (20 mg kg^−1^, i.p.).

**Group 16**

liriodendrin-rich fraction (fraction 4) (40 mg kg^−1^, i.p.).

### 2.8. Statistical Analysis

Results were expressed as mean ± standard error of mean (SEM). The significance of difference between mean was determined by one-way ANOVA test followed by Tukey's; test except the mortality data which was subjected to chi-square test and the results were regarded as significant at *P* < .05.

## 3. Results

### 3.1. Extraction and Preliminary Phytochemical Screening

The yield of the crude methanolic extract (fraction 1) was found to be 10.8% w/w. The yield of fraction 2 of the methanolic extract, that is, chloroform fraction was 1.32% w/w, fraction 3 (phenolic compound fraction) was 0.07% w/w and liriodendrin-rich fraction was 1.4% w/w. Preliminary phytochemical screening showed the presence of alkaloids, carbohydrates, reducing sugars, phenolic compounds, tannins, flavonoids, amino acids and saponins in the crude methanolic extract. In case of different fractions obtained from the crude methanolic extract: fraction 2 showed the presence of alkaloids, carbohydrates, amino acids and flavonoids, fraction 3 showed the presence of phenolic compounds and fraction 4 revealed the presence of carbohydrates and amino acids (Table 1).

### 3.2. PTZ-Induced Convulsions

Anti-convulsant studies with *B. diffusa* extract showed a significant protection against PTZ-induced convulsions in a dose-dependent manner. There was a significant (*P* < .05) delay in the onset of convulsions and reduction in mortality at all the three doses of crude extract (fraction 1) (1000, 1500 and 2000 mg kg^−1^) in PTZ model, as compared with vehicle control group. The different fractions from the crude methanolic extract, that is, fraction 2 (20 mg kg^−1^), fraction 3 (1 mg kg^−1^) and fraction 4 (10, 20 and 40 mg kg^−1^) were also tested in PTZ model for their possible anti-convulsant activity. Fractions 2 and 3 showed no protection against PTZ-induced convulsions even at higher doses (data not shown) and complete mortality was observed in all of these groups. Among the different fractions only fraction 4 showed a significant (*P* < .05) delay in the onset of convulsions and reduced mortality at all the three doses of extract (10, 20 and 40 mg kg^−1^) in PTZ model in a dose-dependent manner showing maximum protection at 40 mg kg^−1^ dose. The activity of liriodendrin-rich extract when combined with sub-effective dose of diazepam (1 mg kg^−1^) showed synergistic effect as the effect of combined treated group was significantly better than either diazepam (4 mg kg^−1^) or liriodendrin-rich fraction (40 mg kg^−1^) *per se* treatment (Table 2).

### 3.3. BAY k-8644-Induced Seizures

**Figure 2:**
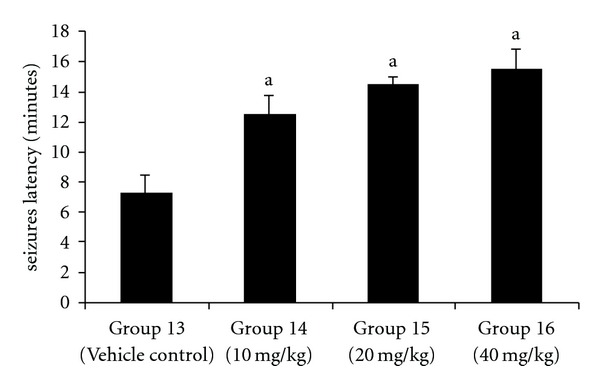
Effect of liriodendrin-rich fraction on the latency of seizures induced by BAY k-8644. ^a^ *P* < .05 as compared with vehicle control group.

**Figure 3:**
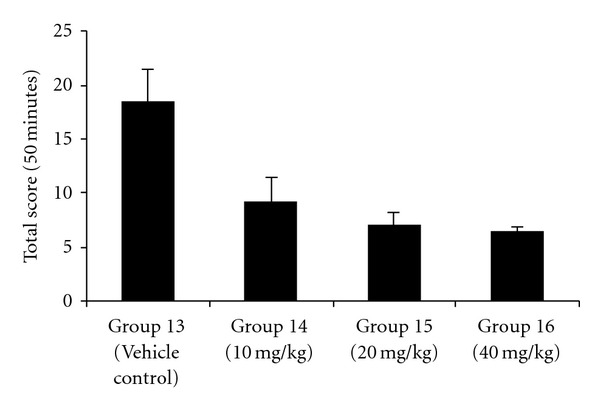
Effect of liriodendrin-rich fraction on the severity of seizures induced by BAY k-8644. ^*a*^ *P* < .05 as compared with vehicle control group.

Pretreatment with liriodendrin-rich fraction showed protection against BAY k-8644-induced seizures. Liriodendrin-rich fraction-treated groups showed a significant (*P* < .05) delay in the onset (Figure 2) and reduction in the severity (Figure 3) of seizures induced by BAY k-8644 in a dose-dependent manner as compared with vehicle control group.

## 4. Discussion

In the present study PTZ was used to induce convulsions owing to the role of calcium ion as a common mediator in PTZ-induced convulsions [22]. This model was suitable for testing our hypothesis that the anti-convulsant activity of *B. diffusa* roots might be due to calcium channel blocking effect by liriodendrin. A significant protection was observed at all the three dose levels of crude methanolic extract of *B. diffusa* roots reiterating its earlier reports of anti-convulsant activity. Therefore the crude methanolic extract was further fractionated to obtain different fractions of methanolic extract rich in different phytoconstituents. Based on the presence of different phytoconstituents in different fractions of the methanolic extract we tried to investigate the component responsible for its anti-convulsant action. As reported, chloroform fraction of the methanolic extract contains fatty acids, sterol glycosides, syringaresinol mono-*β*-d-glucopyranoside and flavonoids [16]. Based on the presence of flavonoids in chloroform fraction it was hypothesized that the activity of methanolic extract might be because of this fraction. As it has been well reported in literature that flavonoids enhance gamma amino butyric acid (GABA) neurotransmission and GABA is the main inhibitory neurotransmitter which is suppressed in epilepsy [23]. Most of the anti-epileptic drugs act by enhancing GABA neurotransmission. It is well documented that in PTZ-induced convulsion model, PTZ exerts its convulsive effect by inhibiting the activity of GABA at GABA_A_ receptors [24] that is why chloroform fraction was the first one among other fractions of methanolic extract to be tested for its anti-convulsant action. But this fraction failed to show any significant protection indicating that the anti-convulsant activity of *B. diffusa* is not because of flavonoids present in chloroform fraction.

The third fraction of the extract containing phenolic compounds was also evaluated in PTZ-induced convulsions with the notion that some phenolic compounds like maltol and other polyphenolic compounds have exhibited anti-epileptic activity [25]. However, this fraction too failed to show protection indicating that the anti-convulsant activity of *B. diffusa* is not because of phenolic compounds present in the methanolic extract.

Finally with pretreatment of fraction 4, that is, liriodendrin-rich fraction, a maximum of 80% protection was observed, which was in accordance with the anti-convulsant activity of crude methanolic extract. Liriodendrin has been reported as a calcium channel blocker [16] and several studies indicate that dihydropyridine (calcium channel antagonists) has anti-convulsant properties [26, 27]; therefore the anti-convulsant action of liriodendrin-rich fraction may be due to its calcium channel antagonistic activity. In concordance with the earlier reports of calcium channel antagonist showing synergistic action with diazepam [28], in the present study diazepam synergized anti-convulsant activity of liriodendrin-rich fraction, thus indicating *B. diffusa* as a good adjuvant to conventional anti-epileptic drug therapy.

**Figure 4:**
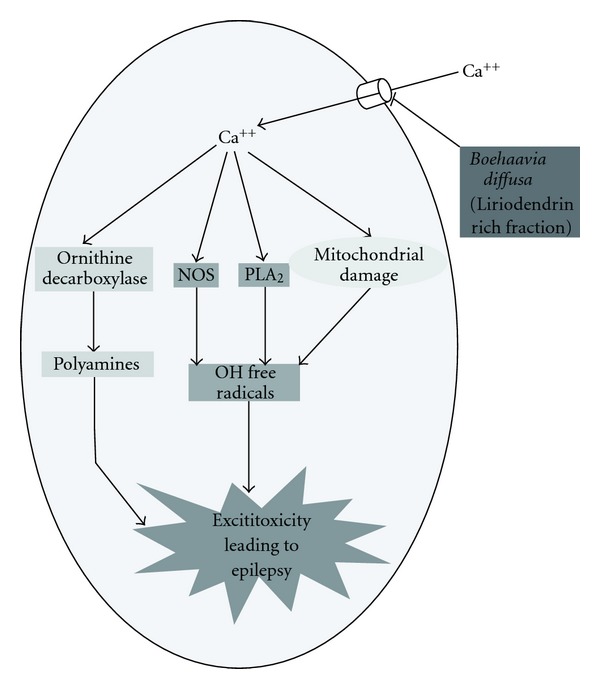
Schematic representation of anti-convulsant mechanism of *B. diffusa*.

Attenuation of seizures induced by BAY k-8644 finally confirmed the role of calcium antagonism in the anti-convulsant activity of liriodendrin-rich fraction of *B. diffusa,* thus approving our hypothesis (Figure 4).

## 5. Conclusions

The findings of the present study suggested that the methanolic extract of *B. diffusa* roots had anti-convulsant activity against PTZ-induced convulsions. As this activity retained only in liriodendrin-rich fraction, this confirms that the anti-convulsant activity of the crude methanolic extract is due to the presence of liriodendrin. Furthermore, protection of BAY k-8644-induced seizures by liriodendrin-rich fraction substantiates that the activity of liriodendrin is due to its calcium channel antagonistic properties.

## Figures and Tables

**Table 1:** Phytoconstituents in *B. diffusa* methanolic extract and its various fractions.

Phytoconstituents (test)	Crude methanolic extract [1]	Fraction 2	Fraction 3	Fraction 4
Alkaloids				
Hager's test	+	+	−	−
Carbohydrates				
Molisch's test	+	+	−	+
Tannins				
FeCl_3_ test	+	−	−	−
Amino acids				
Ninhydrin test	+	+	−	−
Saponins				
Foam test	+	+	−	−
Flavonoids				
Alkali test	+	+	−	−
Phenolic compounds	+	−	+	−

+: present; −: absent.

**Table 2:** Effect of different fractions of methanolic extract of *B. diffusa* on PTZ-induced convulsions in acute treatment studies.

Treatment/dose (mg kg^−1^ **)**	Onset of convulsions (min) (mean ± SEM)	Number of animals showing convulsions	Severity score (mean ± SEM)	Mortality (%)
Vehicle control (75 mg kg^−1^)	1.21 ± 0.06	6/6	4.00 ± 0.00	100
Diazepam (4 mg kg^−1^)	NC	0/6	0	0
Diazepam (1 mg kg^−1^)	2.13 ± 0.07	6/6	3.66 ± 0.23	66.6
Crude extract (1000 mg kg^−1^)	3.97 ± 0.11^a^	4/6	3.66 ± 0.21	66.6
Crude extract (1500 mg kg^−1^)	4.28 ± 0.06^a^	3/6	3.33 ± 0.33	50
Crude extract (2000 mg kg^−1^)	11.33 ± 0.46^*a*^	1/6	1.66 ± 0.50^*a*^	16.6^*a*^
Chloroform fraction (20 mg kg^−1^)	2.10 ± 0.10	5/6	3.83 ± 0.17	83.3
Phenolic compound fraction (1 mg kg^−1^)	1.53 ± 0.05	6/6	4.0 ± 0.00	100
Liriodendrin-rich fraction (10 mg kg^−1^)	2.49 ± 0.19^a^	4/6	3.33 ± 0.33	66.6
Liriodendrin-rich fraction (20 mg kg^−1^)	4.20 ± 0.52^a^	2/6	2.00 ± 0.63^a^	33.3^a^
Liriodendrin-rich fraction (40 mg kg^−1^)	3.07 ± 0.05^a^	0/6	1.83 ± 0.48^a^	0
Liriodendrin-rich fraction (20 mg kg^−1^) + Diazepam (1 mg kg^−1^)	NC	0/6	0	0

^a^ *P* < .05 as compared with vehicle control group.
